# Decision-making styles during stressful scenarios: The role of anxiety in COVID-19 pandemic

**DOI:** 10.3389/fpsyt.2023.1105662

**Published:** 2023-04-05

**Authors:** Mariana Castro Marques da Rocha, Leandro Fernandes Malloy-Diniz, Marco Aurélio Romano-Silva, Rui Mateus Joaquim, Alexandre Luiz de Oliveira Serpa, Alexandre Paim Diaz, Jonas Jardim de Paula, Danielle Souza Costa, Antônio Geraldo da Silva, André Luiz de Carvalho Braule Pinto, Débora Marques de Miranda

**Affiliations:** ^1^SAMBE - Instituto de Saúde Mental Baseada em Evidências, Rio de Janeiro, Brazil; ^2^Post Graduation Department in Molecular Medicine, Universidade Federal de Minas Gerais, Belo Horizonte, Brazil; ^3^Department of Psychiatry, Universidade Federal de Minas Gerais, Belo Horizonte, Brazil; ^4^Department of Pediatrics, Universidade Federal de Minas Gerais, Belo Horizonte, Brazil

**Keywords:** decision making, trait anxiety, post traumatic symptoms, decision strategies, psychological distress, COVID-19

## Abstract

**Introduction:**

Decision-making is not purely rational but highlighted by the influence of intuitive and emotional processes. Recently, researchers have focused more attention on understanding which environmental and personal features influence decision-making processes, and how.

**Objective and methods:**

On this study, we investigate whether Trait Anxiety moderates the impact of Post-Traumatic Stress (PTS) symptoms reported during COVID-19 pandemic on decision-making styles.

**Results:**

The study included 1,358 Brazilian participants (80% women) aged between 20 and 74 (M = 41.11; SD = 11.23) who responded to an online survey between May and August of the year 2021 of COVID-19 pandemic to The State–Trait Anxiety Inventory, The Decisions Styles Scale, The Impact of Event Scale – Revised and questions related to COVID-19. Through moderation analysis, we observed that experiencing PTS is associated with a higher tendency to biased/heuristic decision-making processes.

**Discussion:**

Trait Anxiety seems to influence how people respond to PTS symptoms on decision-making related processes. Subjects with higher Trait Anxiety reported lower tendency to appeal to rationality, especially under higher reported levels of PTS. Meanwhile, lower Trait Anxiety subjects exhibited more reason-based decision-making under higher rates of PTS. This work contributes to a deeper understanding of the interplay among environmental and individual differences on decision-making styles and helps to identify factors of vulnerability for poorer cognitive functioning on stressful scenarios.

## Highlights

- Anxious individuals report lower levels of rationality and intuition on decision-making.
- Post-traumatic symptomatology is associated with more intuitive decision-making strategies.
- Individuals with higher Trait Anxiety tend to be less rational decision-makers under higher display of post-traumatic symptoms.
- Meanwhile, people with lower Trait Anxiety tend to display higher levels of rationality when higher rates of post-traumatic stress are presented.
- Anxiety trait might be considered a vulnerability factor for cognitive functioning in contexts of post-traumatic symptomatology.

## 1. Introduction

On decision-making theories’ crib, choices were described as the result of a conscious, deliberative, process in which known alternatives and their consequences would be analyzed and compared. According to classical theories of decision-making, people chose the most advantageous available option and considered the availability of their own resources (e.g., budget) to handle the consequences of their choices (1, 2). Later, theories started to acknowledge that human behavior, including choice behavior, was not purely rational and highlighted the influence of intuitive and emotional processes (2–7), a proposition now widely accepted.

A growing body of research has proposed people present personal tendencies to rely on either rational or intuitive processes when making decisions. This tendency may be described as “the typical manner by which individuals make decisions” and it is argued that it is built upon personality traits and habit-based inclinations (8). Intuitive and rational decision styles are not mutually exclusive (9) and may work together to provide better outcomes (8). Each one of them presents pros and cons that might be better suited for different contexts. To Payne (10), the efficacy and the expression of each decision style depends on contextual characteristics, in a way that personal tendencies be overrode by situational specificities, leading an individual to be more intuitive than usual, for example.

It is well established that decision-making is influenced by both environmental/contextual factors and individual differences, but, so far, researchers have focused on the study of direct/main effects rather than looking into interactions among them. On this paper, we seek to investigate whether Post-Traumatic Stress (PTS) symptoms reported during COVID-19 pandemic in Brazil impacts on measures of rational and intuitive decision-making styles. Post-Traumatic Stress Disorder (PTSD) is the most frequent and the most investigated psychiatric disorder on population exposed to disasters (11). Several studies associates PTSD to deficits on cognitive performance (12–19), including decision-making (20, 21). Neuroimaging studies suggest that brain regions associated with decision-making are sensitive to changes induced by stress response and behavioral research supports the hypothesis that stress influences decision processes [for a review, see (22)]. Uncertainty related to an unknown disease coursing with socioeconomic implications and frequent deaths seem to amplify the risk perception. So, COVID-19 pandemic can be characterized as a period higher than typical rates of perceived risk (23, 24) and psychological distress (23, 25–28). The rates of post traumatic symptoms and disorders in mixed populations reached 15% of prevalence during SARS and the COVID-19 pandemic including the health care professionals in Brazil (29, 30).

Trait Anxiety (TA) belongs to the neuroticism x emotional stability personality trait and “refers to the stable tendency to attend to, experience, and report negative emotions such as fears, worries and anxiety across many situations” (31: p. 1,989). Hartley and Phelps (32) point out that Pre-Frontal Cortex (PFC)-dependent cognitive and affective functions may be impaired in anxiety disorders. One of the impaired functions would be decision-making since anxiety and decision-making ability share neural substrates. Additionally, PFC activity may reduce susceptibility to biases and promote more rational decision-making (5, 33), which supports the hypothesis that more anxious individuals could make less rational choices. Studies that investigate the relationship between decision-making and anxiety point out that anxious individuals tend to have greater risk aversion, showing preference for safer choices in contexts of uncertainty. They would also exhibit a more pessimistic assessment of the situation (34). Other studies also suggest that anxiety is associated with higher levels of loss aversion (35, 36). As pointed by (37), the relationship between TA and decision-making only recently received attention and little is known about the impact of anxiety on specific decision-making variables (32).

Here we analyze whether TA affects the relationship between PTS symptoms and decision-making during COVID-19 pandemic. Building upon the literature about the impact of stress response on cognitive functioning, and considering TA’s cognitive characteristics, we hypothesize that more anxious individuals might display different patterns of response to PTS symptoms on decision-making strategies when compared to less anxious people. Specifically, we predict that TA might be associated with the use of more intuitive and less rational decision-making strategies.

## 2. Materials and methods

### 2.1. Participants

This study was part of a larger longitudinal online survey, which was approved by the National Commission of Ethics in Research (CONEP) on May 2nd, 2020 (CAAE #: 30823620.6.0000.5149). The recruitment was made through “capture” promotions managed by the Brazilian Psychiatry Association and directed to people across the whole country. On total, 3,341 subjects agreed to participate. Of them, 1,390 declared to have lived a traumatic life event and were included on this study’s sample. Finally, 32 subjects were excluded because they did not completely fill out the questions that assessed this study’s variables of interest or because they failed to inform information regarding sex and age. Data was collected from May to August 2021. The final sample consisted of 1,358 subjects. Sample characterization is presented on Table 1.

**Table 1:** Demographic characteristics.

	Total sample (*n* = 1,358)			Low trait anxiety (*n* = 721)			High trait anxiety (*n* = 637)		
	Mean (SD)	*n* (%)	Min–max	Mean (SD)	*n* (%)	Min–max	Mean (SD)	*n* (%)	Min–max
Sex									
*Male*		271 (20.0)			186 (25.8)			85 (13.3)	
*Female*		1,087 (80.0)			535 (74.2)			552 (86.7)	
Age	41.11 (11.234)		20–74	44.39 (11.307)		21–74	37.40 (9.928)		20–67
Post-traumatic stress	3.17 (2.913)		0–12	2.12 (2.251)		0–12	4.35 (3.118)		0–12
Trait anxiety	14.19 (4.187)		0–24	10.91 (2.245)		0–14	17.90 (2.385)		15–24
Decision style scale (DSS)									
Rational style	20.26 (3.253)		5–25	20.80 (2.908)		10–25	19.65 (3.508)		5–25
Intuitive style	15.10 (3.496)		5–25	15.10 (3.666)		5–25	15.10 (3.296)		5–25
COVID associated distress									
*Directly associated*		120 (8.8)			54 (7.5)			66 (10.4)	
*Indirectly associated*		59 (4.3)			23 (3.2)			36 (5.7)	
*Not associated*		1,097 (80.8)			616 (85.4)			481 (75.5)	
*Not able to respond*		70 (5.2)			21 (2.9)			49 (7.7)	

### 2.2. Instruments

*Impact of Event Scale - Revised (IES-R)*: was created to provide a more complete assessment of responses to traumatic events, being able to cover domains that the Impact of Event Scale (IES) did not yet cover (38). The IES-R is a self-report likert-type scale in which the individual answers the questions based on the 7 days prior to the application of the scale. The Brazilian Portuguese version scale consists of 22 items distributed into 3 subscales: avoidance, intrusion and hyperarousal that include the post-traumatic stress disorder assessment criteria published in DSM-IV [(39), p. 598]. For this study, IES-R shows reliability good reliability using McDonald’s omega 0.96 for Intrusion, 0.93 for avoidance, and 0.94 for hyperarousal.

*State–Trait Anxiety Inventory* (STAI-T): is an instrument developed to measure anxiety across different cultures (40), and was originally created by Spielberger et al. (41). It consists of two subscales: one for state anxiety (STAI-S, item example: “I am tense; I am worried”) and another for trait anxiety (STAI-T, item example: “I worry too much over something that really does not matter”) (40). In the case of our research, we opted for the STAI-T version, which has 20 items in Brazilian Portuguese and is based on a 4-point likert scale (40). On our sample, STAI-T shows a McDonald’s omega of 0.87.

*Decisions Styles Scale* (*DSS*) is a self-report instrument developed by Hamilton et al. (8) adapted to the Brazilian context by Mouta et al. (42). DSS assess two decision-making styles: rational (guided by a deliberative and conscious assessment of options’ pros and cons; item example: “I prefer to gather all the necessary information before committing to a decision”) and intuitive (based on quick and automatic processes, such as “gut” feeling; item example: “When making decisions, I rely mainly on my gut feelings”). The scale consists of 10 items, which are answered on a 5-point likert scale. For our study, DSS shows a McDonalds omega of 0.88 for rational style, and 0.81 for intuitive style.

Participants were asked to inform their biological sex and date of birth, from which their age (in years) were calculated. Participants were also asked to indicate whether their traumatic life event was related to the COVID-19 pandemic, according to the following options: (a) directly associated with COVID-19 pandemic, (b) indirectly associated with COVID-19 pandemic, (c) not associated with COVID-19 pandemic, and (d) not able to answer.

### 2.3. Statistical procedures

First, simple linear regressions were conducted for each one of the dependent variables (rational decision-making style and intuitive decision-making style). Predictors (PTS and TA) were entered separately on individual models to verify each predictor’s main effect. Then, moderation analysis (model 1) was run using Process v3.5 by Hayes (43). Variables were entered as follows: PTS was entered as x (predictor), TA was entered as w (moderator) and decision-making styles (both intuitive and rational) were entered, on distinct analysis, as y (dependent variable). All analysis were run using SPSS 20th version, and we used *p* < 0.05 as a cut-off.

## 3. Results

Demographic data is presented in Table 1, consisted of mostly by women in middle age reporting stress not related to COVID-19.

To test the hypothesis that decision-making style varies as a function of multiple factors and, more specifically, whether trait anxiety moderates the relationship between PTS and decision-making, simple linear regression and simple moderator analysis were conducted. In the first step, a simple linear regression was calculated to predict rational decision-making style based on PTS (Table 2, model 1). A significant regression equation was found [*F*(1,1,356) = 10.752, *p* = 0.001; *R^2^* = 0.008]. The results of the regression indicated that PTS significantly predicted rational decision-making style (*β* = −0.089, p = 0.001). Then, a simple linear regression was run to verify whether rational decision-making style varied as a function of TA (Table 2, model 2). Results indicated that TA significantly predicted rational decision-making style (*β* = −0.176, *p* < 0.001), accounting for approximately 3% of the variance on rational decision-making style reports [*F*(1,1,356) = 43.531, *p* < 0.001].

**Table 2:** Simple regression analysis predicting rational and intuitive decision-making style.

Decision making style	Model	Predictor	*b*	*b*	*β*	*t*	*p*	*R* ^2^
				95% CI				
				(LL–UL)				
Rational style	1	*Constant*	20.571	[20.316; 20.825]		155.317	**<0.0001**	
		Post-traumatic stress	−0.099	[−0.158; −0.040]	−0.089	−3.279	**<0.0001**	0.008
	2	*Constant*	20.796	[20.562; 21.030]		174.307	**<0.0001**	
		Trait anxiety	−1.149	[−1.49; −0.808]	−0.176	−6.598	**<0.0001**	0.030
Intuitive style	3	*Constant*	14.647	[14.374; 14.920]		105.238	**<0.0001**	
		Post-traumatic stress	0.143	[0.079; 0.206]	0.119	4.414	**<0.0001**	0.014
	4	*Constant*	15.096	[14.840; 15.351]		115.903	**<0.0001**	
		Trait anxiety	0.006	[−0.367; 0.379]	0.001	0.033	0.973	0.000

The bold indicates significant at *p*<0.05.

A similar process was conducted for the intuitive decision-making variable. Simple linear regression results (Table 2, model 3) suggest PTS is a valid predictor of intuitive decision-making style (*β* = 0.119, *p* < 0.001) on a model where [*F*(1,1,356) = 19.481, *p* < 0.001, *R*^2^ = 0.014]. Another simple linear regression equation was tested to verify whether intuitive decision-making style varied as a function of TA (Table 2, model 4). The equation was not significant [*F*(1,1,356) = 0.001, *p* = 0.973] and TA was not pointed as a significant predictor of intuitive decision-making style (*β* = 0.001, *p* = 0.973).

**Figure 1:**
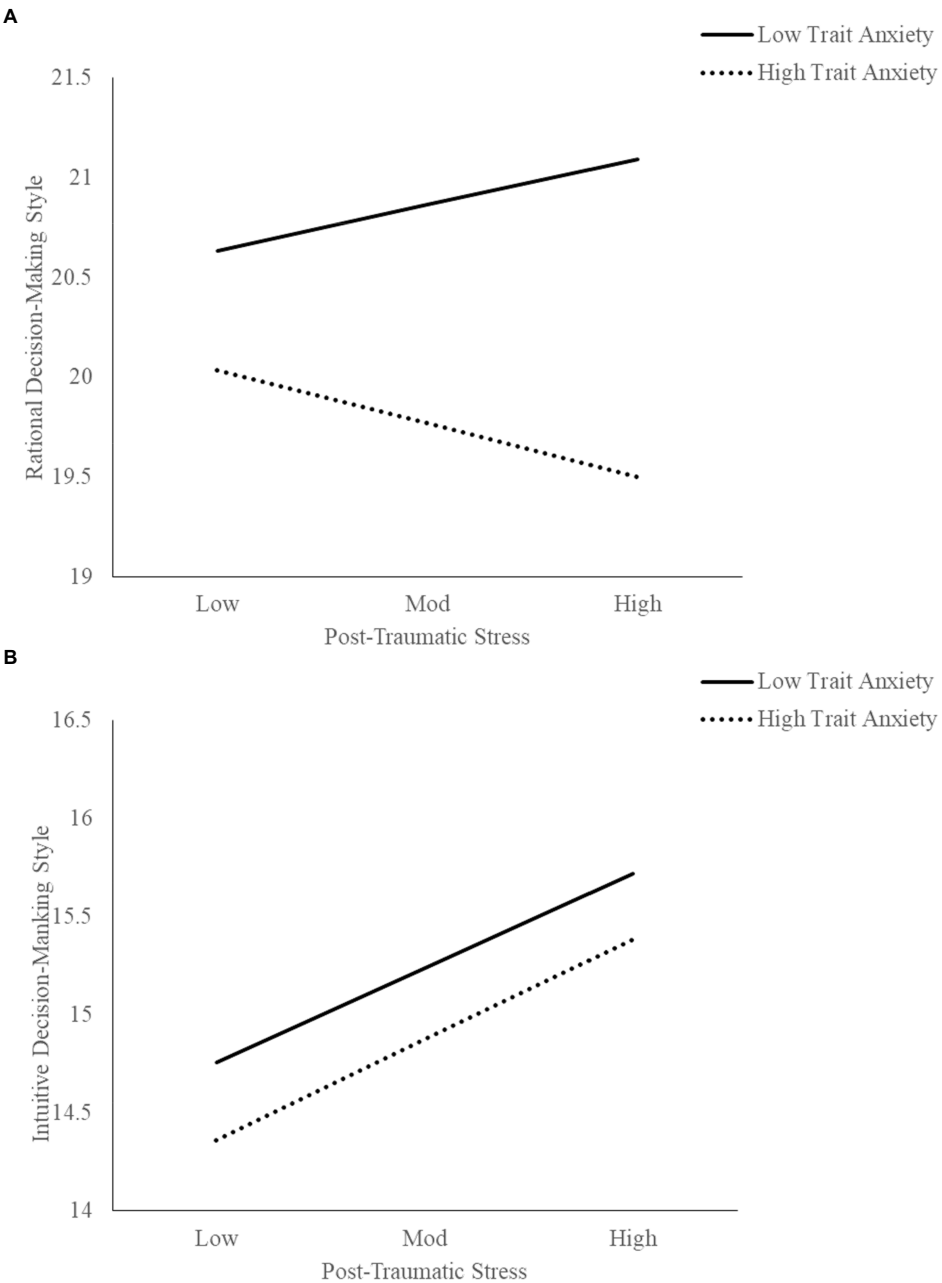
Decision-making style as a function of trait anxiety and post-traumatic stress. **(A)** Corresponds to rational decision-making style and **(B)** corresponds to intuitive decision-making style. Low and high levels of Trait Anxiety were set according to the median value displayed by the sample: Low Trait Anxiety contemplates values under the median value and High Trait Anxiety covers values equal or higher than the median value.

A simple moderator analysis performed to investigate conditional effects of PTS on rational decision-making style (Table 3) showed that the interaction between PTS and TA was statistically significant (*b* = −0.1654, 95% C.I. = −0.2964, −0.0344, *p* < 0.05). According to the results, the conditional effect of PTS on rational decision-making style was only significant when TA was high (TA scale score ≥ 15), with effect = −0.0891, (95% C.I. = −0.1689, −0.0093, *t* = −2.1907, *p* < 0.05). When TA was low (TA scale score < 15), (conditional effect was 0.0763, 95% C.I. = −0.0276, 0.1802, *t* = 1.4412, *p* = 0.1479). These results suggest TA acts as a negative moderator of the relationship between PTS and rational decision-making style. Conditional effects for rational decision-making are represented on Figure 1A. Results also suggest PTS’ impact on rational decision-making is only significant when TA is high.

**Table 3:** Results from a regression analysis examining the moderation of the effect of PTS on rational and intuitive decision-making style by trait anxiety.

Decision making style	Predictor	*b*	*b*	SE B	*T*	*p*
			95% CI			
			(LL, UL)			
Rational Style	Constant	208.761	[20.6183; 21.1338]	0.1314	1.588.936	**<0.0001**
	Post-traumatic stress	0.0763	[−0.0276; 0.1802]	0.0530	14.412	0.1497
	Trait anxiety	−11.236	[−1.4940; −0.7533]	0.1888	−59.518	**<0.0001**
	Post-traumatic stress*Trait anxiety	−0.1644	[−0.2964; −0.0344]	0.0668	−24.774	**0.0134**
Intuitive style	Constant	152.632	[14.9834; 15.5429]	0.1426	1.070.358	**<0.0001**
	Post-traumatic stress	0.1598	[0.0471; 0.2726]	0.0575	27.808	**0.005**
	Trait anxiety	−0.3639	[−0.7658; 0.0381]	0.2049	−17.759	0.076
	Post-traumatic stress*Trait anxiety	0.0111	[−0.1310; 0.1533]	0.0725	0.1538	0.878

For rational style *R*^2^ = 0.036, MSE = 10.2262, *F*(3,1,354) = 16.8546, *p* < 0.001; for intuitive style *R*^2^ = 0.0165, MSE = 12.0464, *F*(3,1,354) = 7.5792, *p* < 0.001.

The bold indicates significant at *p*<0.05.

Results from the simple moderator analysis performed to investigate conditional effects of PTS on intuitive decision-making style (Table 3) showed that the interaction between PTS distress and TA was not statistically significant (*b* = 0.0111, 95% C.I. = −0.1310, 0.1533, *p* = 0.8778). Regression analysis with mean-centered variables showed that only PTS was a significant main predictor of intuitive decision-making (*b* = 0.1598, 95% C.I. = 0.0471, 0.2726, *t* = 2.7808, *p* = 0.0055). There was a trend for the main effect of TA on intuitive decision-making (*b* = −0.3639, 95% C.I. = −0.7658, 0.0381, *t* = −1.7759, *p* = 0.076). These results suggest no moderation effect of TA on the relationship between PTS and intuitive decision-making style. Effects for intuitive decision-making are visually displayed at Figure 1B.

Taken together, results suggest the direction of the impact of PTS on rational decision-making style depends on TA level.

## 4. Discussion

This study investigated the relationship between Post-Traumatic Stress and decision-making styles, and whether Trait Anxiety moderates it. Our findings suggest that the impact of exposure to stressful contexts, measured by PTS, may change how people make decisions. Furthermore, the direction of this change depends on how vulnerable to anxiety people are. People who reported higher TA exhibited lower levels of both rational and intuitive decision-making styles when compared to subjects who reported lower tendency to anxiety. More importantly, on contexts of trauma, the more anxious people adopted a less rational decision-making style while subjects with lower TA displayed more reason-based strategies.

Some mechanisms might be behind the observed results. The first one is cognitive overload hypothesis. Literature on decision-making suggests that, in uncertain scenarios, our mental resources, necessary for self-control, are drained, which leads to an increase of the preference for “wants” over “shoulds” (44). The cognitive overload hypothesis is also present in the reasoning used to explain why people exposed to contexts of poverty, debt and other stressful scenarios have worse cognitive functioning when compared to other populations and themselves in less challenging contexts (45, 46). TA might compromise the ability to use effort-based strategies under stress *via* cognitive depletion due to heightened mental activity toward anxiety-influenced processes, such as worry, catastrophizing and planning for hypothetical scenarios.

Uncertainty is also a factor that might influence the observed results. With the beginning of the COVID-19 pandemic, the world experienced the biggest periods of uncertainty of the recent history. In Brazil, the scenario is aggravated by factors such as government misinformation through contradictory directions by authorities responsible for coordinating actions to deal with the current health crisis (47). Brazilians were therefore immersed in uncertainties that encompass the economic, political, social, and health spheres. We suggest it is plausible to consider that the general context of uncertainty might play a part on how people experienced stress response. In a study of two different stressful scenarios, such as in medical activities after earthquake and in COVID-19, a population also composed mostly by women perceive stress as anxiety, somatization, and depression acutely. The organization and participation on work related to the response to the event were important to perception of distress to improve health and wellbeing of professionals (48).

According to our analysis, PTS was positively associated to a more intuitive decision-making style. This observation is consistent with the findings that suggest uncertainty elicits intuitive/automatic processes of decision-making (22). It is possible to argue that TA’s heightened vulnerability to uncertainty might be part of the mechanism through which PTS decreases the use of analytical/rational decision-making strategies on high TA individuals.

Another point that might be helpful on the interpretation of our results concerns the heightened sensitivity to internal sensation associated to the experience of emotions exhibited by high TA individuals (49). Baradell and Klein (50) conducted a study in which subjects with higher inner body consciousness showed increased susceptibility to experience impact of critical life events and daily struggles on decision-making. It is possible to argue that TA increases subjective perception of stress through sensitivity to emotion-related inner body sensations, therefore modulating the impact of distress on decision-making processes.

One of the greatest limitations of this study is the conceptualization on decision-making styles and its influence on results interpretation. While some authors understand decision-styles as crystalized constructs (primarily defined by personality traits and, therefore, constant throughout life), others highlight the influence of habit-based learning on personal tendencies on how to approach decision tasks. However, as pointed earlier, authors believe decision styles might be influenced by acute contextual factors, such as time pressure (7, 10). Our work tries to attend earlier recommendation to try identifying such factors. Finally, this study presents limitations regarding its sample. Subjects who participated on this research were self-selected and had to be able to assess the internet, which might create a sampling bias toward subjects with higher socioeconomic condition.

In conclusion, this work results suggest that people with higher tendency to display more frequent, intense, and dysfunctional levels of anxiety may suffer greater cognitive impact on stressful scenarios. Our findings align with and may contribute to the theory of differential sensitivity to context, which proposes that people with different levels of environmental vulnerability experience different outcomes when exposed to stress (51). Our work might contribute to the understanding of how stress impacts cognitive functioning and help to identify the most vulnerable individuals.

## Data availability statement

The raw data supporting the conclusions of this article will be made available by the authors, without undue reservation.

## Ethics statement

The studies involving human participants were reviewed and approved by Universidade Federal de Minas Gerais - CONEP. The patients/participants provided their written informed consent to participate in this study.

## Author contributions

MR: designed the study, wrote the draft, revised literature, and analyzed data. LM-D, AS, and DM: supervision, work on project design and search for funding. RJ, AS, APa, JP, DC, and MR-S: help on data collect and study design and discussions. APi: analyzed data and revise the manuscript. All authors contributed to the article and approved the submitted version.

## Funding

This study was funded by governmental funding by the agencies: CAPES, CNPq, OPAS, MS-Brasil, and FAPEMIG.

## Conflict of interest

The authors declare that the research was conducted in the absence of any commercial or financial relationships that could be construed as a potential conflict of interest.

## Publisher’s note

All claims expressed in this article are solely those of the authors and do not necessarily represent those of their affiliated organizations, or those of the publisher, the editors and the reviewers. Any product that may be evaluated in this article, or claim that may be made by its manufacturer, is not guaranteed or endorsed by the publisher.

## References

[1] Gigerenzer G Gaissmaier W. Decision making: nonrational theories In: . International encyclopedia of the social & behavioral sciences. Amsterdam: Elsevier (2015)

[2] Von Neumann J Morgenstern O. Theory of games and economic behavior. Bull Amer Math Soc. (1945) 51:498–5. doi: 10.1090/S0002-9904-1945-08391-8

[3] Damasio H Grabowski T Frank R Galaburda AM Damasio AR. The return of Phineas gage: clues about the brain from the skull of a famous patient. Science. (1994) 264:1102–5. doi: 10.1126/science.8178168, PMID: 8178168

[4] Kahneman D. A perspective on judgment and choice: mapping bounded rationality. Am Psychol. (2003) 58:697–08. doi: 10.1037/0003-066X.58.9.697, PMID: 14584987

[5] De Martino B Kumaran D Seymour B Dolan RJ. Frames, biases, and rational decision-making in the human brain. Science. (2006) 313:684–7. doi: 10.1126/science.1128356, PMID: 16888142 PMC2631940

[6] Kahneman D Tversky A. Prospect theory: an analysis of decision under risk In: MacLean LC Ziemba WT, editors. Handbook of the fundamentals of financial decision making: Part I. Singapore: World Scientific (2013)

[7] Bechara A Damasio H Damasio AR. Role of the amygdala in decision-making. Ann N Y Acad Sci. (2003) 985:356–9. doi: 10.1111/j.1749-6632.2003.tb07094.x, PMID: 12724171

[8] Hamilton K Shih SI Mohammed S. The development and validation of the rational and intuitive decision styles scale. J Pers Assess. (2016) 98:523–5. doi: 10.1080/00223891.2015.1132426, PMID: 26967981

[9] Sadler-Smith E. The intuitive style: relationships with local/global and verbal/visual styles, gender, and superstitious reasoning. Learn Individ Differ. (2011) 21:263–08. doi: 10.1016/j.lindif.2010.11.013

[10] Payne JW. Contingent decision behavior. Psychological Bulletin. (1982) 92:382–402. doi: 10.1037/0033-2909.92.2.382, PMID: 14584987

[11] Galea S Nandi A Vlahov D. The epidemiology of post-traumatic stress disorder after disasters. Epidemiol Rev. (2005) 27:78–91. doi: 10.1093/epirev/mxi003 15958429

[12] Brandes D Ben-Schachar G Gilboa A Bonne O Freedman S Shalev AY. PTSD symptoms and cognitive performance in recent trauma survivors. Psychiatry Res. (2002) 110:231–8. doi: 10.1016/S0165-1781(02)00125-7, PMID: 12127473

[13] Burri A Maercker A Krammer S Simmen-Janevska K. Childhood trauma and PTSD symptoms increase the risk of cognitive impairment in a sample of former indentured child laborers in old age. PLoS One. (2013) 8:e57826. doi: 10.1371/journal.pone.0057826, PMID: 23469076 PMC3582641

[14] Leskin LP White PM. Attentional networks reveal executive function deficits in posttraumatic stress disorder. Neuropsychology. (2007) 21:275–4. doi: 10.1037/0894-4105.21.3.275, PMID: 17484590

[15] Qureshi SU Long ME Bradshaw MR Pyne JM Magruder KM Kimbrell T. Does PTSD impair cognition beyond the effect of trauma? J Neuropsychiatry Clin Neurosci. (2011) 23:16–28. doi: 10.1176/appi.neuropsych.23.1.16, PMID: 21304135

[16] Twamley EW Allard CB Thorp SR Norman SB Cissell SH Berardi KH. Cognitive impairment and functioning in PTSD related to intimate partner violence. J Int Neuropsychol Soc. (2009) 15:879–7. doi: 10.1017/S135561770999049X, PMID: 19703324

[17] Roos LE Knight EL Beauchamp KG Berkman ET Faraday K Hyslop K. Acute stress impairs inhibitory control based on individual differences in parasympathetic nervous system activity. Biol Psychol. (2017) 125:58–63. doi: 10.1016/j.biopsycho.2017.03.004, PMID: 28268165 PMC5448703

[18] Roozendaal B. Stress and memory: opposing effects of glucocorticoids on memory consolidation and memory retrieval. Neurobiol Learn Mem. (2002) 78:578–5. doi: 10.1006/nlme.2002.4080, PMID: 12559837

[19] Vedhara K Hyde J Gilchrist ID Tytherleigh M Plummer S. Acute stress, memory, attention and cortisol. Psychoneuroendocrinology. (2000) 25:535–9. doi: 10.1016/S0306-4530(00)00008-1 10840167

[20] Morgado P Sousa N Cerqueira JJ. The impact of stress in decision making in the context of uncertainty. J Neurosci Res. (2014) 93:839–7. doi: 10.1002/jnr.23521, PMID: 25483118

[21] Sailer U Robinson S Fischmeister FP Konig D Oppenauer C Lueger-Schuster B. Altered reward processing in the nucleus accumbens and mesial prefrontal cortex of patients with posttraumatic stress disorder. Neuropsychologia. (2008) 46:2836–44. doi: 10.1016/j.neuropsychologia.2008.05.022 18597797

[22] Starcke K Brand M. Decision making under stress: a selective review. Neurosci Biobehav Rev. (2012) 36:1228–48. doi: 10.1016/j.neubiorev.2012.02.003, PMID: 22342781

[23] Petzold MB Bendau A Plag J Pyrkosch L Mascarell Maricic L Betzler F. Risk, resilience, psychological distress, and anxiety at the beginning of the COVID-19 pandemic in Germany. Brain Behav. (2020) 10:e01745. doi: 10.1002/brb3.1745, PMID: 32633464 PMC7361063

[24] Wise T Zbozinek TD Michelini G Hagan CC Mobbs D. Changes in risk perception and self-reported protective behaviour during the first week of the COVID-19 pandemic in the United States. R Soc Open Sci. (2020) 7:200742. doi: 10.1098/rsos.200742, PMID: 33047037 PMC7540790

[25] Breslau J Finucane ML Locker AR Baird MD Roth EA Collins RL. A longitudinal study of psychological distress in the United States before and during the COVID-19 pandemic. Prev Med. (2021) 143:106362. doi: 10.1016/j.ypmed.2020.106362, PMID: 33388325 PMC9753596

[26] Gómez-Salgado J Andrés-Villas M Domínguez-Salas S Díaz-Milanés D Ruiz-Frutos C. Related health factors of psychological distress during the COVID-19 pandemic in Spain. Int J Environ Res Public Health. (2020) 17:3947. doi: 10.3390/ijerph17113947, PMID: 32498401 PMC7312369

[27] Kikuchi H Machida M Nakamura I Saito R Odagiri Y Kojima T. Changes in psychological distress during the COVID-19 pandemic in Japan: a longitudinal study. J Epidemiol. (2020) 30:522–8. doi: 10.2188/jea.JE20200271, PMID: 32963212 PMC7557175

[28] Twenge JM Joiner TE. Mental distress among US adults during the COVID-19 pandemic. J Clin Psychol. (2020) 76:2170–82. doi: 10.1002/jclp.23064, PMID: 33037608 PMC7675251

[29] Pinto B Carvalho AL Serpa AL Jardim J de Paula D Costa S. Increased risk of health professionals to feel traumatized during the COVID-19 pandemic. Sci Rep. (2021) 11:18286. doi: 10.1038/s41598-021-97783-6, PMID: 34521958 PMC8440540

[30] Cheung T Cheng CPW Fong TK Sharew NT Anders RL Xiang YT. Psychological impact on healthcare workers, general population and affected individuals of SARS and COVID-19: a systematic review and meta-analysis. Front Public Health. (2022) 10. doi: 10.3389/fpubh.2022.1004558, PMID: 36407973 PMC9673757

[31] Gidron Y. Trait Anxiety. In: Gellman MD, Turner JR, editors. Encyclopedia of Behavioral Medicine. New York, NY: Springer Science+Business Media (2013).

[32] Hartley CA Phelps EA. Anxiety and decision-making. Biol Psychiatry. (2012) 72:113–8. doi: 10.1016/j.biopsych.2011.12.027, PMID: 22325982 PMC3864559

[33] Xu P Gu R Broster LS Wu R Van Dam NT Jiang Y. Neural basis of emotional decision making in trait anxiety. J Neurosci. (2013) 33:18641–53. doi: 10.1523/JNEUROSCI.1253-13.2013, PMID: 24259585 PMC3834062

[34] Maldonato M Dell’Orco S. How to make decisions in an uncertain world: heuristics, biases, and risk perception. World Futures. (2011) 67:569–7. doi: 10.1080/02604027.2011.615591

[35] De Martino B Camerer CF Adolphs R. Amygdala damage eliminates monetary loss aversion. Proc Natl Acad Sci USA. (2010) 107:3788–92. doi: 10.1073/pnas.0910230107, PMID: 20142490 PMC2840433

[36] Sokol-Hessner P Hsu M Curley NG Delgado MR Camerer CF Phelps EA. Thinking like a trader selectively reduces individuals' loss aversion. Proc Natl Acad Sci USA. (2009) 106:5035–40. doi: 10.1073/pnas.0806761106, PMID: 19289824 PMC2656558

[37] Miu AC Heilman RM Houser D. Anxiety impairs decision-making: Psychophysiological evidence from an Iowa Gambling Task. Task Biol Psychol. (2008) 77:353–8. doi: 10.1016/j.biopsycho.2007.11.010, PMID: 18191013

[38] Weiss DS. The impact of event scale — revised In: Wilson JP Keane TM, editors. Assessing psychological trauma and PTSD. 2nd ed. New York: The Guilford Press (2004).

[39] Caiuby AVS Lacerda SS Quintana MI Torii TS Andreoli SB. Adaptação transcultural da versão Brasileira da Escala do Impacto do Evento - Revisada (IES-R) [cross-cultural adaptation of the Brazilian version of the event impact scale - revised (IES-R)]. Cad Saude Publica. (2012) 28:597–3. doi: 10.1590/S0102-311X2012000300019, PMID: 22415191

[40] Fioravanti-Bastos ACM Cheniaux E Landeira-Fernandez J. Development and validation of a short-form version of the Brazilian state-trait anxiety inventory. Psicologia: Reflexão e Crítica. (2011) 24:485–4.

[41] Spielberger CD Gorsuch RC Lushene RE. Manual for the state trait anxiety inventory. Palo Alto, CA: Consulting Psychologists Press (1970).

[42] Mouta GS Pinto AB Malloy-Diniz LF Pasian RS. Cross-cultural adaptation, and factor structure of the decision styles scale for Brazil. Curr Res Behav Sci. (2021) 2:100039. doi: 10.1016/j.crbeha.2021.100039

[43] Hayes AF. Introduction to mediation, moderation, and conditional process analysis: A regression-based approach. New York: Guilford publications (2017).

[44] Milkman KL. Unsure what the future will bring? You may overindulge: uncertainty increases the appeal of wants over should. Organ Behav Hum Decis Process. (2012) 119:163–6. doi: 10.1016/j.obhdp.2012.07.003

[45] Mani A Mullainathan S Shafir E Zhao J. Poverty impedes cognitive function. Science. (2013) 341:976–08. doi: 10.1126/science.1238041, PMID: 23990553

[46] Ong Q Theseira W Ng IY. Reducing debt improves psychological functioning and changes decision-making in the poor. Proc Natl Acad Sci USA. (2019) 116:7244–9. doi: 10.1073/pnas.1810901116, PMID: 30910964 PMC6462060

[47] Xavier DR E Silva EL Lara FA E Silva GR Oliveira MF Gurgel H. Involvement of political and socio-economic factors in the spatial and temporal dynamics of COVID-19 outcomes in Brazil: a population-based study. Lancet Region HealthAmericas. (2022) 10:100221. doi: 10.1016/j.lana.2022.100221 PMC8918677 35309089

[48] Ciuffini R Marrelli R Leuter C Stratta P Paladini A Ciccozzi A. The stress response of intensive care unit medical doctors facing repeated severe emergencies. Front Psychol. (2022) 13:895954. doi: 10.3389/fpsyg.2022.895954, PMID: 36506986 PMC9730870

[49] Pollatos O Traut-Mattausch E Schroeder H Schandry R. Interoceptive awareness mediates the relationship between anxiety and the intensity of unpleasant feelings. J Anxiety Disord. (2007) 21:931–3. doi: 10.1016/j.janxdis.2006.12.004, PMID: 17257810

[50] Baradell JG Klein K. Relationship of life stress and body consciousness to hypervigilant decision making. J Pers Soc Psychol. (1993) 64:267–3. doi: 10.1037/0022-3514.64.2.267

[51] Boyce WT Ellis BJ. Biological sensitivity to context: I. an evolutionary–developmental theory of the origins and functions of stress reactivity. Dev Psychopathol. (2005) 17:271–1. 16761546 10.1017/s0954579405050145

